# The Neuroepigenetic Landscape of Vertebrate and Invertebrate Models of Neurodegenerative Diseases

**DOI:** 10.1177/25168657221135848

**Published:** 2022-11-04

**Authors:** Thanga Harini Sundaramoorthy, Isabel Castanho

**Affiliations:** 1 University of Exeter Medical School, University of Exeter, Exeter, UK; 2 Beth Israel Deaconess Medical Center, Boston, MA, USA; 3 Harvard Medical School, Boston, MA, USA

**Keywords:** Neurodegenerative diseases, neurodegeneration, model organisms, neuroepigenetics, DNA methylation, histone modifications, miRNA, Alzheimer’s disease, Parkinson’s disease, amyotrophic lateral sclerosis

## Abstract

Vertebrate and invertebrate models of neurodegenerative diseases, such as Alzheimer’s disease, Parkinson’s disease, and amyotrophic lateral sclerosis, have been paramount to our understanding of the pathophysiology of these conditions; however, the brain epigenetic landscape is less well established in these disease models. DNA methylation, histone modifications, and microRNAs are among commonly studied mechanisms of epigenetic regulation. Genome-wide studies and candidate studies of specific methylation marks, histone marks, and microRNAs have demonstrated the dysregulation of these mechanisms in models of neurodegenerative diseases; however, the studies to date are scarce and inconclusive and the implications of many of these changes are still not fully understood. In this review, we summarize epigenetic changes reported to date in the brain of vertebrate and invertebrate models used to study neurodegenerative diseases, specifically diseases affecting the aging population. We also discuss caveats of epigenetic research so far and the use of disease models to understand neurodegenerative diseases, with the aim of improving the use of model organisms in this context in future studies.

## Introduction

Neurodegenerative diseases comprise a group of chronic neurological disorders characterized by progressive functional and structural neuronal deterioration, ultimately resulting in death.^1^ Some of the most common neurodegenerative diseases causing increasing morbidity and mortality in the aging population are Alzheimer’s disease (AD),^2^ Parkinson’s disease (PD)^3^ and amyotrophic lateral sclerosis (ALS).^4^ Model organisms displaying aspects of these diseases have enabled the understanding of some of the molecular mechanisms underpinning their pathologic etiologies as well as the course of neurodegenerative processes.^5^ Increasing research has shown changes in genomic regulatory markers and machinery in human post-mortem brains from people with these conditions, which may play a role in disease pathogenesis; however, genomic changes are less well established in model organisms that offer the advantage of investigating early stages of disease as opposed to post-mortem end stages, as well as manipulating therapeutic targets.^6-8^ This review aims to explore genomic regulatory changes reported to date in the brain in model organisms used to study neurodegenerative diseases, and address caveats of their use in neuroepigenetic research, in order to promote better and a more targeted use of disease models.

## Pathophysiology, Etiology, and Genetics of Neurodegenerative Diseases

AD, PD, and ALS are each characterized by specific pathological brain features that can only be definitively confirmed post-mortem. These hallmark features include amyloid-β (Aβ) plaques and tau neurofibrillary tangles in AD, α-synuclein aggregates in PD, and inclusions of TAR DNA-binding protein-43 (TDP-43) in ALS. The progression of these individual pathologies is disease-specific, and each can be found in various different regions of the brain; however, regions primarily affected by these pathological features, and hence commonly studied in disease models, include the hippocampus and the cerebral cortex in AD, the substantia nigra in PD, and the motor cortex, brainstem, and spinal cord in ALS.^1^

The causes of these neurodegenerative conditions are both familial and sporadic in nature. Familial forms are often early-onset (<65 years) due to genetic predisposition. For example, autosomal dominant mutations in the amyloid precursor protein (*APP*), presenilin 1 (*PSEN1*) or presenilin 2 (*PSEN2*) genes result into familial AD; mutations in the genes encoding α-synuclein (*SNCA*) or leucine rich repeat kinase 2 (*LRRK2*) cause autosomal-dominant PD; mutations in the superoxide dismutase 1 (*SOD1*) gene result into familial ALS.^1,9^ Notably, these genetic mutations can be introduced in animals to model disease phenotypes, which will be discussed later in this review.

**Figure 1.**
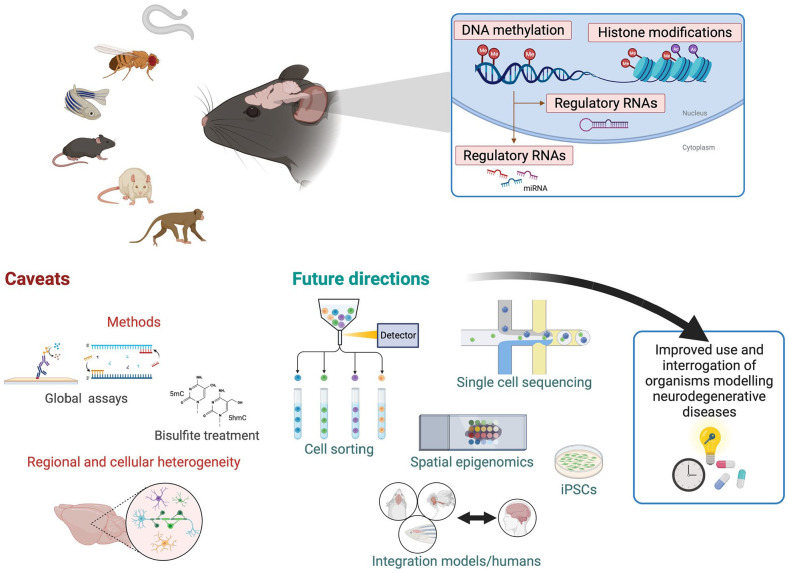
Vertebrate and invertebrate models useful for epigenetics research of neurodegerative diseases, epigenetic marks investigated to date, common caveats, and future directions to improve the use and benefits of these powerful models. Model organisms used to study neuroepigenetics of neurodegenerative diseases (top left) range from simpler organisms such as nematodes (*Caenorhabditis elegans*), fruit fly (*Drosophila melanogaster*) and zebra fish (*Danio rerio*), rodents (mice and rats), and non-human primates. DNA methylation, histone modifications and regulatory RNAs (predominantly miRNAs) comprise epigenetic processes that have been studied in these models (top right). Caveats of studies to date (bottom left) include methods commonly employed, many assessing global levels of epigenetic modifications (global assays), and—in the case of DNA methylation—the vast majority relying in bisulfite conversion, which does not allow to differentiate between 5-methylcytosine (5mC) and 5-hydroxymethylcytosine (5hmC). Of great importance in epigenetics research, regional and cellular heterogeneity have predominantly not been taken into account and/or explored. Future research (bottom right) must mitigate limitations of studies thus far by discerning tissue- and cell-specific signatures, take advantage of immerging single-cell and spatial technologies, integrate findings from model organisms with human studies, and consider additional strategies such as the parallel use of iPSCs. Taken together with the development of better disease models currently underway, future research should aim to improve the use of model organisms for the understanding of epigenetic processes in neurogenerative conditions, and of how and when we should modify and manipulate aspects of human disease, particularly important for drug discovery and testing. Created with BioRender.com.

The aforementioned autosomal dominant inheritance accounts for a minor proportion of disease incidence for AD, PD and ALS, however; the vast majority of cases are late-onset (>65 years) and sporadic.^10^ Nevertheless, the existence of a genetic component contributing to sporadic forms is widely accepted given the scientific evidence of recent years, especially propelled by genome-wide association studies that have reported how genetic variation may increase (or decrease) disease susceptibility.^1,10^ Additionally, studies investigating monozygotic twins (ie, individuals that share the same genetic information) have shown discordance in the onset and progression of these conditions,^11-15^ suggesting that the genetic contribution to disease etiology and progression goes beyond the genetic sequence, likely including epigenetic mechanisms involved in genomic regulation and expression. DNA methylation, histone modifications and non-coding RNAs are among the most commonly recognized epigenetic mechanisms (Figure 1) associated with enhancing or silencing gene expression, and have been shown to play crucial roles in neurogenesis and early brain development.^16^ Importantly, changes to these regulatory mechanisms and their associated molecular machinery are observed in post-mortem brain tissue from people exhibiting neurodegenerative diseases, as reviewed by others.^6-8^

Research investigating the role of epigenetic processes in the context of neurodegenerative diseases has exponentially increased in recent years, aiming to understand its implications, and particularly pushed forward with the advancement of technologies facilitating the study of epigenetic changes.

## Vertebrate and Invertebrate Models in Neuroscience Research

Vertebrate and invertebrate organisms are distinguished by the presence and absence of a vertebral column, respectively. They are characterized by unique genetic makeups, which give rise to their individual embryonic and developmental processes. Mutual molecular, biological and genomic features between these species exist, however, that make them valuable tools for neuroscience research, including for the study of human neurodegenerative diseases.^17^ There are currently no animal species that can precisely model the complexity of the human nervous system nor its associated disease states; nevertheless, animal models recapitulating aspects of neurodegenerative disease have been developed, allowing to investigate molecular, biological, and pathological mechanisms, with potential translation to humans.

Several limitations and ethical issues make carrying out brain research in humans very challenging. Human brain tissue used to study neurodegenerative diseases is usually collected post-mortem, often presenting significant brain damage as a consequence of late stages of disease, making it difficult to get a perspective that goes beyond the final stages of disease and understand causal factors and changes that occurred over disease course. The use of animal models hence helps overcoming these challenges by providing more flexibility and facilitating the study of early and progressive changes.

Rodents (eg, mice and rats) and non-human primates are the most popularly used vertebrates to model neurodegenerative diseases. Mice in particular can be easily genetically modified to express disease-specific genetic mutations, thereby phenocopying familial forms and exhibiting disease-associated pathologies. Genetic manipulation can also be performed on rats but to a lesser extent due to reduced feasibility. In addition, rodents can also be chemically induced to recapitulate disease, for example through injections of artificial pathological proteins or extracts from post-mortem human brain tissue. This type of approach results in cognitive impairment in rodent models, consistent with functional impairments seen in the neurodegenerative human brain. More recently, spontaneous models have also been described, where pathology is driven primarily by aging, constituting a promising strategy to recapitulate sporadic forms of disease.^18^ For instance, the senescence-accelerated mouse prone 8 (SAMP8) model is used to model sporadic AD and is driven primarily by accelerated aging. The model exhibits Aβ and tau pathology as well as cognitive impairments, characteristic of AD.^19^ Table 1 lists the mouse models of AD reported in this review. Non-human primates, such as monkeys, have many similarities with humans due to their biological proximity, behavioral complexity, and the development of natural neurodegenerative pathology, such as the accumulation of Aβ plaques. This makes them an advantageous model; however, their long lifespan and ethical concerns restrict their use for the study of neurodegenerative diseases.^17^

**Table 1.** Mouse models of Alzheimer’s disease reported in this review.

Model	Description
3xTg-AD	*APP* Swedish, *MAPT* P301L, *PSEN1* M146V mutations^20^
5xFAD	*APP* Swedish, Florida, and London mutations, and *PSEN1* M146L and L286V mutations^21^
APP^NL-F^	*APP* Swedish and Iberian mutations^22^
APP^NL-G-F^	*APP* Swedish, Arctic, and Iberian mutations^22^
APP_SWE, IND_	*APP* Swedish and Indiana mutations (under Thy1 promoter)^23^
APP/PS1	*APP* Sweden and *PSEN1* ΔE9 mutations^24^
APP/PS1-21	*APP* Swedish and *PSEN1* L166P mutations^25^
APP23	*APP* Swedish double mutations (K651M and N652L)^26^
CK-p25	Overexpression of p25 (a regulator of cyclin-dependent kinase)^27^
J20	*APP* Swedish and Indiana mutations (under PDGF-β promoter)^28^
OA42i	Oligomeric amyloid β_1–42_ plus ibotenic acid (oA42i) induced mouse^29^
P301S	*MAPT* P301S mutation^30^
PSAPP	*APP* Swedish and *PSEN1* M146L mutations^31^
PSEN dKO	Deletions of *PSEN1* and *PSEN2*^32^
SAMP8	Senescence accelerated mouse developed from AKR/J natural mouse line^33^
SAMP10	Senescence accelerated mouse developed from AKR/J natural mouse line^34^
Tau-22	*MAPT* G272V and P301S mutations^35^
Tg2576	*APP* Swedish mutation^36^

Lower vertebrates, such as *Danio rerio* (zebrafish), have seen an increase in popularity as valuable models in neuroscience in recent years. Some advantages for their use include being small, and thus requiring relatively-simple and small research facilities, the fact that they grow at a fast rate, and how easily they can be genetically manipulated; however, despite exhibiting a DNA methylation system similar to mammals, the absence of gene orthologs for many human genes limits their use.^18,37^ Invertebrates such as *Drosophila melanogaster* (fruit fly) and *Caenorhabditis elegans* (nematode worm) also share many similarities with humans at the molecular level. These organisms have a fully sequenced genome and can also be easily genetically modified; however, their physiology is very different to that of humans, and their DNA methylation profiles are distinct to those in humans, therefore limiting their use as disease models for the study of epigenetics in neurodegenerative disorders.^17^

## DNA Methylation

DNA methylation, the most commonly studied epigenetic mark, regulates gene expression, generally by promoting gene silencing. It involves the covalent transfer of a methyl group from S-adenosyl methionine onto the fifth carbon atom of cytosine nucleotides on DNA. This modified state is known as 5-methylcytosine (5mC) and can be found concentrated at cytosine-guanine-rich regions (or CpG islands) in the DNA. Further modifications can also occur: 5mC can be converted 5-hydroxymethylcytosine (5hmC), 5-formylcytosine (5fC), and 5-carboxylcytosine (5caC).^16^ 5hmC, in particular, was originally thought to be a transient state, later shown to have an important role on its own, particularly in the brain.^38^ Of note, as mentioned, DNA modifications present differently in distinct organisms used for the study of neurodegenerative disorders. For example, non-CpG DNA methylation is restricted to vertebrates.^39^ In *C. elegans* in particular, DNA methylation was initially considered to be absent, but was later shown to be restricted to adenine N6-methylation (6mA).^40^

Various studies have investigated changes in global 5mC and 5hmC levels in mouse models of AD; however, the findings reported to date are not consistent between studies (Table 2).^23,41-48^ Whilst study design caveats may be one reason for this discordance, such as the assessment of a small number of samples, the type of model used in each study may also be a contributing factor. Another important factor that contributes to the complexity of these findings includes differences across distinct brain regions and/or stages of a disease. The brain regions reported to exhibit changes in global 5mC and 5hmC include the hippocampus and the cerebral cortex, which are primarily affected in AD, suggesting vulnerability of pathology-affected brain regions to changes in these methylation marks. In contrast, findings from the cerebellum showed no changes in 5mC and 5hmC in multiple genetic AD mouse models.^23,41,48^ This parallels the fact that the cerebellum is often spared of pathological changes in the human AD brain,^49^ and is consistent with DNA methylation studies in humans.^50^

**Table 2.** DNA methylation and associated machinery in mouse models of Alzheimer’s disease, Parkinson’s disease, and amyotrophic lateral sclerosis.

Regulation	Brain region	Mouse model	Change	Reference
Alzheimer’s disease				
5mC	Hippocampus	APP_Swe, Ind_	↓	Do Carmo et al^23^
		5xFAD	–	Zhang et al^41^, Griñán-Ferré et al^42^, Griñán-Ferré et al^43^, Griñán-Ferré et al^44^
		SAMP8	↑	Cosín-Tomás et al^45^
	Cerebral cortex	APP_Swe, Ind_	↓	Do Carmo et al^23^
		APP/PS1	↑	Huang et al^46^
		3xTg-AD	–	Cadena-del-Castillo et al^47^
	Prefrontal cortex	3xTg-AD	–	Zhang et al^41^
	Cerebellum	APP_Swe, Ind_	–	Do Carmo et al^23^
		3xTg-AD	–	Zhang et al^41^
5hmC	Hippocampus	APP/PS1	↓	Shu et al^48^
		3xTg-AD	↓	Zhang et al^41^
		5xFAD	–	Griñán-Ferré et al^42^, Griñán-Ferré et al^43^, Griñán-Ferré et al^44^
		SAMP8	↑	Cosín-Tomás et al^45^
	Cerebral cortex	3xTg-AD	↑	Cadena-del-Castillo et al^47^
		APP/PS1	↑	Huang et al^46^
		APP/PS1	–	Shu et al^48^
	Prefrontal cortex	3xTg-AD	↓	Zhang et al^41^
	Cerebellum	3xTg-AD	–	Zhang et al^41^
		APP/PS1	–	Shu et al^48^
DNMT1	Hippocampus	5xFAD	↓	Griñán-Ferré et al^42^
		5xFAD	–	Griñán-Ferré et al^44^
		SAMP8	↓	Cosín-Tomás et al^45^
DNMT3a	Hippocampus	5xFAD	↑	Griñán-Ferré et al^42^
		5xFAD	–	Griñán-Ferré et al^44^
		SAMP8	↓	Cosín-Tomás et al^45^
DNMT3b	Hippocampus	5xFAD	↑	Griñán-Ferré et al^42^, Griñán-Ferré et al^44^
		SAMP8	–	Cosín-Tomás et al^45^
TET1	Hippocampus	5xFAD	↓	Griñán-Ferré et al^42^, Cosín-Tomás et al^45^
		5xFAD	↑	Griñán-Ferré et al^44^
		3xTg-AD	↓	Zhang et al^41^
	Prefrontal cortex	3xTg-AD	↓	Zhang et al^41^
TET2	Hippocampus	3xTg-AD	↓	Zhang et al^41^
		SAMP8	↓	Cosín-Tomás et al^45^
		5xFAD	–	Griñán-Ferré et al^42^
		5xFAD	↑	Griñán-Ferré et al^44^
	Prefrontal cortex	3xTg-AD	↓	Zhang et al^41^
TET3	Hippocampus	3xTg-AD	↓	Zhang et al^41^
	Prefrontal cortex	3xTg-AD	↓	Zhang et al^41^
MeCP2	Hippocampus	APP/PS1	↑	Lu et al^62^
	Striatum	APP/PS1	↓	Li et al^63^
Parkinson’s disease				
5mC	Anterior brain cortex	α-synuclein	↓	Desplats et al^51^
DNMT1	Anterior brain cortex	α-synuclein	↓	Desplats et al^51^
	Substantia nigra	MPTP-induced	↓	Zhang et al^64^
TET2	Substantia nigra	MPTP-induced	↑	Wu et al^65^
Amyotrophic lateral sclerosis				
5mC	Global brain	SOD1_G93A_	–	Figueroa-Romero et al^52^
5hmC	Global brain	SOD1_G93A_	↑	Figueroa-Romero et al^52^

Abbreviations: 5mC, 5-methylcytosine; 5hmC, 5-hydroxymethylcytosine; DNMT, DNA methyltransferase; MeCP, methyl-CpG-binding protein; TET, ten-eleven translocation enzyme family.

The up arrow (↑) represents upregulation or increased levels. The down arrow (↓) represents downregulation or decreased levels. The dash (-) represents no significant changes identified.

Only a few studies to date have investigated DNA methylation in models of PD and ALS (Table 2). In a mouse model of PD that overexpresses human α-synuclein, Desplats et al^51^ reported reduced global 5mC levels in the anterior portion of the brain compared to control mice. In a mouse model of ALS bearing the *SOD1* G93A mutation, Figueroa-Romero et al^52^ identified increased 5hmC global levels compared to control mice. Human post-mortem studies investigating brain tissue from individuals with PD^53-55^ and ALS^15,56-58^ have revealed aberrant methylation in pathology-affected brain regions. Future studies using mouse models should investigate these changes in pathology-affected brain regions, such as the substantia nigra in PD.

DNA 6mA (methylation of the sixth carbon atom of adenine nucleotides) and RNA N6-methyladenosine (m6A) have also been identified important epigenetic modifications in recent years,^59,60^ although their functions are still to be clearly defined. Interestingly, a recent study reported reduced DNA 6mA levels in peripheral blood in people with AD when compared to controls, demonstrating its potential as a biomarker.^61^ Further studies exploring DNA 6mA and RNA m6A are needed, especially studies aiming to understand their role in the brain and how they change in and with disease.

Contrarily to cancer, DNA methylation fluctuations in the brain are much more subtle, thus methods assessing global levels of DNA methylation may not be the most appropriate in this context, which may explain the disparity of reports to date discussed in this section. Quantitative interrogation of selected sites (eg, methylation arrays) and at single-base resolution (eg, next generation sequencing)—which will be considered in the next section—can provide much more valuable insights. Especially when coupled with methodologies involving cell-sorting or single-cell sequencing, these powerful approaches can provide more insightful information, disclose heterogeneity, and likely clarify discordances observed in previous studies.

### Genome-wide DNA methylation profiling

In recent years, methylation arrays and sequencing techniques have made feasible DNA methylation analyses on a more extensive—genome-wide—level in both humans and animal models. Of interest, some studies performing genome-wide analyses of mouse models of AD have been conducted using diverse techniques (Table 3).^41,48,66-70^ Whole-genome bisulfite sequencing (WGBS) is considered the gold standard approach for studying genome-wide methylation at the single-base resolution as it profiles methylation patterns in the entire methylome at both CpG islands and non-CpG regions.^71^ Zhang et al^68^ used this method to investigate the genome-wide DNA methylation profile in the cerebral cortex of SAMP8 mice and reported higher methylation levels in introns compared to exons. Three differentially-methylated regions (DMRs) annotated to *Dlgap1*, *Eif2ak2*, and *Tmem51* showed increased methylation levels in SAMP8 mice in this study, likely associated with AD pathogenesis.^68^ Zhang et al and colleagues further investigated differential expression of these genes, and reported that *Eif2ak2* expression was downregulated due to increased methylation, while *Dlgap1* and *Tmem51* expression was upregulated.^68^ These findings emphasize the complexity and diversity of the functional roles of DNA methylation; although DNA methylation is often associated with transcriptional repression, this is not always the case. Not to mention that there is currently an emerging interest in understanding the role of DNA methylation in the regulation of alternative splicing.^72^

**Table 3.** Genome-wide DNA methylation analyses studies of mouse models of Alzheimer’s disease.

Study	Model	Brain tissue	Modification	Method	Main findings
Sanchez-Mut et al^66^	APP/PS1 (12 mo) 3xTg-AD (18 mo)	Twelve brain regions, including CA1, CA3 and DG hippocampal subregions, frontal cortex, and hypothalamus	5mC	MeDIP sequencing, Illumina VeraCode GoldenGate DNA Methylation Mouse Array	Hypermethylation of *Sorbs3*, *Spnb4* and *Tbxa2r* in the prefrontal cortex of APP/PS1 and 3xTg-AD mice consistent with the hypermethylation of *SORBS3*, *SPTBN4* and *TBXA2R* in post-mortem human AD brain tissue
Cong et al^67^	APP/PS1 (11 mo)	Cerebral cortex	5mC	MeDIP sequencing, Roche NimbleGen, Mouse DNA Methylation 3 ×720 K CpG Island Plus RefSeq Promoter Array	2346 hypermethylated regions of which 2221 mapped to promoter regions and 485 genes were associated with AD
Zhang et al^68^	SAMP8 (7 mo)	Cerebral cortex	5mC	WGBS	63 DMRs of which 41 regions were hypermethylated and 22 regions were hypomethylated in SAMP8 mice
Tang et al^69^	PSEN dKO (12 mo)	Hippocampus	5mC	RRBS	1094 hypermethylated regions and 1676 hypomethylated regions in PSEN dKO mice
Kundu et al^70^	APP^NL-G-F^ and APP^NL-F^ (12 mo)	Hippocampus	5mC	RRBS	57 DMRs shared between both AD mouse models
Shu et al^48^	APP/PS1 (12- and 67-wk)	Hippocampus	5hmC	hMeDIP sequencing	Decrease in total peaks in aged APP/PS1 mice
Zhang et al^41^	3xTg-AD (E16.5)	Cortical neurons (fetal brain)	5hmC	hMeDIP sequencing	Decrease in total peaks in AD group, with most 5hmC marks located in intergenic regions

Abbreviations: 5hmC, 5-hydroxymethylcytosine; 5mC, 5-methylcytosine; AD, Alzheimer’s disease; CA1, *cornu ammonis* 1; CA3, *cornu ammonis* 3; DG, dentate gyrus; dKO, double knock-out; DMR, differentially methylated region; MeDIP, methylated DNA-immunoprecipitation; WGBS, whole genome bisulfite sequencing ; RRBS, reduced representation bisulfite sequencing; hMeDIP, hydroxymethylated DNA-immunoprecipitation.

An alternative method to WGBS, that can also be used to investigate DNA methylation at a genome-wide level, is reduced representation bisulfite sequencing (RRBS). Essentially, a reduced, representative sample of the whole genome at single-base resolution is sequenced, mostly enriched by CpG regions.^71^ Two studies employed this technique on AD mouse models and identified differentially-methylated regions in the hippocampus enriched for processes relevant to AD and brain homeostatic mechanisms, such as adhesion signaling, cytoskeleton, and synaptic functions.^69,70^

Research profiling genomic 5hmC have also been conducted in AD mouse models, although still in its infancy.^41,48^ Two studies used hydroxymethylated DNA-immunoprecipitation sequencing (hMeDIP-seq) to identify changes in 5hmC. Both studies showed a reduction in overall 5hmC in the respective tissues investigated, and identified specific enrichment of differentially-hydroxymethylated regions annotated to genes related to synaptic and neuronal homeostasis, as well as AD pathogenesis.^41,48^ This suggests that alongside 5mC changes, 5hmC-mediated regulation may also play a critical role in AD pathogenesis and neurodegeneration.

### Writers, erasers, and readers

An additional viewpoint of studying DNA methylation mechanisms is looking at the (dys)regulation of the machinery responsible for generating and maintaining it. DNA methyltransferases (DNMTs), known as methylation “writers,” add methyl groups to the DNA, giving rise to 5mC. Among the major DNMTs, DNMT1 is considered the maintenance methylase, and DNMT3a and DNMT3b are considered de novo methylases.^16^ DNMT3a and DNMT3b are of particular interest in the context of the topic of this review as they introduce methylation marks to DNA over time; however, studies exploring these enzymes in AD mouse models reported conflicting findings (Table 2).^42,44,45^ For example, studies investigating DNMT1 expression reported either a decrease in hippocampal expression in AD mice,^42,45^ or no significant changes^44^ (Table 2). DNMT1 levels were also shown to be decreased in 2 unique mouse models of PD.^51,64^ It is unclear whether DNMT1 downregulation could be a mutual characteristic across these neurodegenerative diseases and additional studies should investigate this further. DNMT1 is imperative in reproducing DNA methylation after DNA replication and thus helps maintain chromosome stability. A potential consequence for the reported reduction in DNMT1 is a resulting reduction of DNA methylation, that is, hypomethylation in these pathology-affected brain regions.^16^

The family of ten-eleven translocation (TET) enzymes, known as “erasers,” are methylcytosine dioxygenases that convert 5mC to 5hmC. Research has shown conflicting levels of expression of these enzymes across several mouse models of AD in pathology-affected regions (Table 2).^41,42,44,45^ In a mouse model of PD (MPTP-induced, that is, generated by inducing the neurotoxin 1-methyl-4-phenyl-1,2,3,6-tetrahydropyridine or MPTP, which is highly selective for the substantia nigra and leads to dopaminergic neuron damage), a study demonstrated increased *Tet2* gene expression in the substantia nigra compared to control mice.^65^ As for other epigenetic mechanisms discussed earlier, the relevance of these changes in neurodegenerative conditions is still unclear.

Proteins of the methyl-CpG-binding domain (MBD) family, known as “readers,” recognize and bind specific regions of the genome (usually methylated CpG islands) and recruit chromatin-remodeling proteins to induce changes in DNA transcription; however, these are less well researched.^16^ Some of the MBD-recognized genomic sites include promoter regions found upstream of genes where transcription is initiated. Of the very few studies investigating this, Bie et al^73^ and Lu et al^62^ reported increased hippocampal methyl-CpG-binding protein 2 (MeCP2) levels in rodent models of AD. By using a chromatin immunoprecipitation (ChIP) assay, the teams showed increased cytosine methylation and binding of MeCP2 at the promoter region of the gene encoding neuroligin-1 (*Nlgn1*) in the hippocampus of Aβ_1-40_-induced rats^73^ and APP/PS1 mice^62^ compared to their respective controls. Neuroligin-1 plays a critical role at synapses, and its dysfunction has been closely associated with AD pathogenesis. Targeting and increasing the expression of *Nlgn1* to promote its neuroprotective effects at synapses offers a therapeutic opportunity in slowing down neurodegenerative processes.^62,73^ A different study demonstrated reduced MeCP2 levels in the striatum of 3-month-old APP/PS1 mice compared to controls, with specific S421 phosphorylation of MeCP2 shown to be increased in the cytoplasm in neurons. The authors suggested that this finding was due to de novo phosphorylation of MeCP2 after AD pathological injury, possibly acting to relieve transcriptional repression.^63^

The implications of changes in expression of “reader” proteins in the context of neurodegenerative diseases require further investigation.

## Histone Modifications

Epigenetic modifications in histones (the proteins that package the DNA into nucleosomes) regulate gene expression by influencing the structure of chromatin and by controlling the binding of regulatory proteins and other effector molecules.^16^ The core histones that make up the nucleosomes are histones 2A (H2A), 2B (H2B), 3 (H3), and 4 (H4). These can be epigenetically modified through histone modifications to alter chromatin structure and enable chromatin remodeling. Examples of common histone modifications include lysine and arginine methylation, lysine acetylation, and serine and threonine phosphorylation amongst an array of covalent changes.^74^

Histone acetylation is generally associated with active gene expression at transcription start sites, such as acetylation of lysine at the ninth position of H3 (H3K9ac) and lysine at the 27th position of H3 (H3K27ac).^75^ Meanwhile, methylated histone marks are often associated with inactive genes. For instance, tri-methylation of lysine at the ninth position of H3 (H3K9me3) and the 27th position of H3 (H3K27me3) are usually distributed across inactive regions and at transcription start sites of inactive genes, respectively. However, some methylated histone marks can be found at active gene loci (eg, H3K4me and H3K4me3). Histone phosphorylation is not as well characterized; nonetheless, H3S10ph and H3T11ph marks seem to have a role in developmental processes.^75^

Overall, the studies summarized in Table 4 and described in detail in the following sections demonstrate how changes in histone modifications can have detrimental and widespread effects by interfering with the transcription of crucial neuroprotective genes in neurodegenerative conditions.

**Table 4.** Changes to histone marks in animal models of Alzheimer’s disease.

Regulation	Study	Model system (age)	Findings
Histone acetylation	Govindarajan et al^78^	Mouse, APP/PS1-21 (15 mo)	↓ H3K9ac in CA1, CA3, DG, Pir, and M1/2 ↓ H3K14ac in CA3, DG, Pir, Cg and M1/2 ↓ H4K5ac in CA1, CA3, DG, Pir, Cg, and M1/2 ↓ H4K12ac in CA1, CA3, DG and M1/2 ↓ H4K16ac in CA1, CA3, DG
	Zhang et al^79^	Aβ_42_-induced *Drosophila* of early age (third instar larvae) and late age (28-dadults)	↓ H4K16ac in the brain of early and late age models
	Lithner et al^80^	Mouse, Tg2576 (4 mo)	↑ H3K14ac in CA3, CTX, PFC
	Chatterjee et al^81^	Mouse, Tau-22 (12 mo)	↓ H2BK5K10K15K20ac in dHP
	Gräff et al^82^	Mouse, CK-p25 (3-6 mo)	↓ H2BK5ac, H3K14ac, H4K5ac and H4K12ac at multiple genes associated with learning and memory, and synaptic plasticity in HP
	Gjoneska et al^83^	Mouse, CK-p25 (3 mo)	H3K27ac peaks in HP (see text for details)
	Klein et al^84^	Mouse, P301S Tau (6 and 11 mo) Mouse, CK-p25 (3 mo)	H3K9ac peaks in HP (see text for details)
Histone methylation	Lithner et al^80^	Mouse, Tg2576 (4 mo)	↑ H3K9me2 in DG, CTX
	Zheng et al^85^	Mouse, 5xFAD (5-6 month)	↑ H3K9me2 in PFC
	Cao et al^86^	Mouse, P301S Tau (5-6 mo)	↑ H3K4me3 in PFC
	Gjoneska et al^83^	Mouse, CK-p25 (3 mo)	H3K4me3, H3K4me1, H3K27me3, H3K36me3, H4K20me1, H3K9me3 peaks in HP
Histone phosphorylation	Anderson et al^87^	Mouse, 5xFAD (3 mo)	↓ H3S57ph, H3T58ph and both combined in whole mouse brain homogenates

Abbreviations: CA1, *cornu ammonis* 1; CA3, *cornu ammonis* 3; Cg, cingulate cortex; CTX, cerebral cortex; DG, dentate gyrus; dHP, dorsal hippocampus; HP, hippocampus; M1/2, motor cortex 1 and 2; Pir, piriform cortex; PFC, prefrontal cortex.

The up arrow (↑) represents upregulation or an increase in level. The down arrow (↓) represents downregulation or a decrease in level. CA1, CA3 and DG are regions of the hippocampus.

Histone acetylation is generally associated with transcriptional activation since it reduces the affinity of the histone tail for adjacent nucleosomes and relaxes chromatin structure, allowing transcription machinery to bind to the DNA.^75^ Immunoassay techniques have been recurrently used to assess global histone acetylation levels in mouse models of AD.^45,76,77^ One study using this type of approach identified reduced H3 acetylation in 8-month-old SAMP8 mice that was partly restored by exercize.^76^ Another study focused on age-associated changes in the 3xTg-AD mouse and reported higher H3 and H4 acetylation at 4-, 8-, 11-, and 12-months of age, becoming increasingly discordant with age.^77^ Global changes in H3ac and H4ac levels should be carefully interpreted, however, as it is not evident how the acetylation marks are distributed in the genome and, thus, how it is being altered.

Various studies have identified changes in histone acetylation marks in the brain, either in whole brain in one study using flies or in defined brain regions in mice (Table 4).^78-82^ For example, decreased H4K16ac was reported in both mice and flies modeling AD, specifically in hippocampal subregions of APP/PS1-21 mice^78^ and in the brain of young and old aged Aβ_42_-induced *Drosophila*.^79^ Whilst studies investigating regional or global levels of specific histone marks help understand epigenetic changes, it is critical to identify where these marks are enriched in the genome.

Gräff et al^82^ characterized specific histone marks at selected brain regions using a conventional approach. The team performed reverse transcriptase-polymerase chain reaction (RT-PCR) to identify H2BK5ac-, H3K14ac-, H4K12ac-, and H4K5ac-immunoprecipitated chromatin at the promoters of selected neuroplasticity and housekeeping genes in the hippocampus of the CK-p25 mouse model of AD. They found reduced H2BK5ac, H3K14ac, H4K12ac and H4K5ac histone marks at numerous candidate genes involved in learning and memory (eg, *Bdnf*, *Cdk5*) and synaptic plasticity (eg, *GluR1, NR2A, NR2B*).^82^

A study by Gjoneska et al^83^ profiled 7 histone marks (H3K4me3, H3K4me1, H3K27ac, H3K27me3, H3K36me3, H4K20me1, and H3K9me3) in the hippocampus of CK-p25 mice using ChIP-sequencing (ChIP-seq). The team found 3667 increased and 5056 decreased H3K4me3 peaks at active promoter regions, and 2456 increased and 2154 decreased H3K27ac peaks at active enhancer regions in CK-p25 mice. The increased-levels in enhancer and promoter regions were mainly associated with immune and stimulus-response functions, whereas the decreased-levels were principally associated with synapses and learning-associated functions.^83^ Similarly, Marzi et al^88^ performed a histone acetylome-wide association study using ChIP-seq to examine H3K27ac in entorhinal cortex tissues from people with AD, demonstrating extensive variation in H3K27ac across the genome. Some of the hyperacetylated peaks (increased H3K27ac marks) identified were associated with genes related to Aβ and tau pathology and response to hypoxia, whereas some of the hypoacetylated peaks (decreased H3K27ac marks) were associated with genes related to neuronal transmission and synapses.^88^ Notably, these are similar to the associated pathways discussed by Gjoneska et al,^83^ where authors further reported mouse-human conservation of chromatin state profiles identified in enhancers in p25-inducible transgenic mice. Specifically, regions orthologous to increased-level enhancers in mouse exhibited immune cell enhancer activity in humans, and orthologs of decreased-level enhancers in mouse corresponded to fetal brain enhancer activity in humans, suggestive of alterations in regulatory regions involved in neuronal plasticity.

More recently, Klein et al^84^ sought to compare changes in H3K9ac marks between aged human cortices and 2 AD mouse models: MAPT P301S and CK-p25 mice. The study showed spatial patterns in H3K9ac marks that were similar in both AD mouse models and consistent with findings from aged human cortices, reinforcing the utility of these model systems to further explore and modify chromatin regulation changes observed in AD.^84^

In the substantia nigra of a new mouse model of PD, the MitoPark mouse, Huang et al^89^ also found disturbed levels of H3K27ac. The MitoPark mouse model reported in this study was generated by conditionally knocking out mitochondrial transcription factor A in dopaminergic neurons, and expressing multiple features of PD. Huang et al^89^ suggested that hyperacetylation of H3K27 may have arisen due to the mitochondrial dysfunction, that is, generation of the MitoPark mice per se, with the resulting epigenetic changes likely contributing to PD pathogenesis. Further studies are needed for clarification.

Histone methylation is often, but not always, associated with gene and chromatin silencing (eg, H3K9 and H3K27 di- and tri-methylation).^75^ Studies investigating AD mouse models have shown increased H3K9me2 in the cerebral cortex and DG subregion of the hippocampus of Tg2576 mice^80^ and the prefrontal cortex of 5xFAD mice^85^ (Table 4). Post-mortem prefrontal cortex sample from people with AD were also reported to present with higher H3K9me2 compared to healthy controls.^85^ In contrast, studies in AD mouse models so far showed no significant difference in H3K27me3 levels in the prefrontal cortex, 85,86 M1/M2 motor cortex and S1/S2 somatosensory cortex compared to controls.^90^

H3K4me and H3K4me3 are frequently found at promoters of active genes and enhance transcription and gene expression.^75^ A study by Cao et al^86^ reported increased H3K4me3 but not H3K4me in the prefrontal cortex of the P301S Tau mouse model of AD, mirroring their findings from post-mortem prefrontal cortex tissues from AD patients. In contrast, Dyer et al^90^ did not find any significant changes in H3K4me3 levels in the M1/M2 motor cortex and S1/S2 somatosensory cortex of 3-, 6-, and 12-month-old APP/PS1 mice.

Recent research has provided evidence for sex-specific differences in specific histone marks (such as H3K4me3, H3K27ac and H3K27me3) in the cortex of the PSAPP mouse model of AD.^91^ This distribution lies in transcription control regions of genes involved in neuronal functions, which have been associated with cognitive decline in AD patients. Future research should explore whether these changes are also seen in histone writer and eraser enzymes, particularly due to their therapeutical potential.

H3K4me3 has also been implicated in PD models. A study by Nicholas et al^92^ found decreased H3K4me3 in the striatum of MPTP-induced mice and macaque monkeys. A subsequent study demonstrated the restoration of this histone mark in vitro following treatment with a histone demethylase inhibitor that can cross the blood brain barrier.^93^ In 6-OHDA-induced PD rats, the same histone demethylase inhibitor rescued dopaminergic neuron loss and motor defects—characteristic features of PD—demonstrating its potential as a therapeutic agent for the treatment of PD.^93^

Phosphorylation of histones adds a significant negative charge to the nucleosome complex, thereby opening up the chromatin structure. This modification is not as commonly researched as the histone modifications previously discussed. One study, by Anderson et al^87^ reported decreased H3S57 and H3T58 phosphorylation, either separately or in combination, in the 5xFAD mouse brain (Table 4). Investigating the implications of these histone marks on a genomic and transcriptomic level may be more valuable in identifying the downstream effects, but no studies of such nature have been reported to date.

### Histone writers and erasers

A diverse range of enzymes is responsible for the chemical modifications that occur to histones.^74^ The methyl, acetyl and phosphate groups can be added to histones by “writer” enzymes, such as histone methyltransferases (HMT), acetyltransferases and kinases. These enzymes can be further categorized; for instance, HMTs have high selectivity for lysine and arginine residues, hence they are grouped into lysine methyltransferases (KMTs) and arginine methyltransferases. The chemical groups can also be removed from histones by “eraser” enzymes, such as histone demethylases, deacetylases (HDAC), and phosphatases. These enzymes can also be further categorized; for instance, the HDAC family contains 18 HDAC proteins that can be divided into 2 subcategories and 4 classes in total: classical (classes I, II, and IV) and sirtuins (class III).^74^

Several studies have identified increased HDAC2 levels in the hippocampus^45,82,94^ and prefrontal cortex^82,94^ of AD mouse models and an Aβ_42_-induced AD *Drosophila* model^79^ compared to their respective controls (Table 5). Specifically, Gräff et al^82^ showed increased HDAC2 binding localized to specific learning and memory, as well as synaptic plasticity genes in the hippocampus of CK-p25 mice compared to controls. Moreover, studies investigating epigenetic changes in the *Nlgn1* promoter discussed earlier in this review reported increased HDAC2 binding at this region in the hippocampus of Aβ_1-40_-induced rats^73^ and APP/PS1 mice.^62^ Indeed, NLGN1 is a protein associated with synaptic function and transmission.^95^ A mouse model of PD bearing the *LRRK2* R1441G mutation also was also shown to exhibit increased HDAC2 alongside increased HDAC1 and HDAC3 levels.^96^ Meanwhile, other HDACs did not show consistent findings between AD model organisms (Table 5).^29,45,76,82^ Taken together, this suggests that targeting HDAC2 may hold considerable therapeutic potential amongst the other HDACs. Indeed, this modulation of HDAC2 has been recently explored with promising results, with Nakatsuka et al^97^ reporting amelioration of deficits in long-term potentiation and memory impairment in the hippocampus of APP/PS1 mice following HDAC2 inhibition.

**Table 5.** Changes in histone-modifying enzymes in animal models of Alzheimer’s disease and Parkinson’s disease.

Regulation	Reference	Model system (age)	Findings
Alzheimer’s			
Histone deacetylation	Cosin-Tomas et al^45^	Mouse, SAMP8 (2 and 9 mo)	↑ HDAC1 in HP of both early and late age models ↑ HDAC2 in HP of 2-mo-old SAMP8 mouse only ↓ SIRT1 in HP of both early and late age models ↓ SIRT6 in HP of 9-mo-old SAMP8 mouse only
	Zhang et al^79^	Aβ_42_-induced *Drosophila* of early age (third instar larvae) and late age (28-day adults)	↑ HDAC2 in the brain of both early and late age models
	Gräff et al^82^	Mouse, CK-p25 (3-6 mo), 5xFAD (6 mo)	↑ HDAC2 in CA1 and PFC of CK-p25 mouse ↑ HDAC2 in HP and PFC of 5xFAD mouse
	Liu et al^94^	Mouse, 3xTg-AD (12 mo)	↑ HDAC2 in HP
	Cosín-Tomás et al^76^	Mouse, SAMP8 (8 mo)	↓ HDAC5 and HDAC6 in HP ↓ SIRT1 in HP
	ArunSundar et al^29^	Mouse, oA42i-induced (age not stated)	↑ HDAC6 in HP
	Song et al^98^	Mouse, 5xFAD (6 mo)	↓ SIRT1 in HP
	Zhang et al^100^	Mouse, APP/PS1 (6 mo)	↓ SIRT1 in HP
	Rodriguez-Ortiz et al^99^	Mouse, 3xTg-AD (12 mo)	↓ SIRT1 in vHP
Histone methylation	Cao et al^86^	Mouse, P301S Tau (5-6 mo)	↑ KMT2A and SETD1B in PFC
	Zheng et al^85^	Mouse, 5xFAD (5-6 mo)	↑ EHMT1 and EHMT2 in PFC
Parkinson’s			
Histone deacetylation	Kim et al^96^	Mouse, LRRK2 (11 mo)	↑ HDAC1, HDAC2 and HDAC3
	Tao et al^102^	Mouse, Rotenone-induced (8 wk old)	↓ SIRT1 in SN

Abbreviations: CA1, *cornu ammonis* 1; EHMT, euchromatic histone-lysine methyltransferase; HDAC, histone deacetylase; HP, hippocampus; KMT, lysine methyltransferase; PFC, prefrontal cortex; SETD; family of SET-domain methyltransferases; SIRT, sirtuin; SN, substantia nigra; vHP ventral hippocampus.

The up arrow (↑) represents upregulation or an increase in level. The down arrow (↓) represents downregulation or a decrease in level.

Multiple studies demonstrated a decrease in *Sirtuin-1* levels in the hippocampus of a range of AD mouse models (Table 5).^45,76,98-100^ Specifically, the decrease reported in 3xTg-AD mice was restricted to the ventral hippocampus and not observed in the dorsal hippocampus.^99^ The dorsal hippocampus (posterior in primates) is primarily involved in cognitive functions such as learning and memory, whilst the ventral hippocampus (anterior in primates) is associated with emotions and motivation processes; both regions of the brain and their respective functions are affected in AD.^101^ Furthermore, this decrease was only observed in the 12-month-old 3xTg-AD mice but not in the younger mice, suggesting age-associated effects or influence by stage and progression of the disease.^99^

In a mouse model of PD induced by rotenone (a pesticide and complex I inhibitor that reproduces features of PD, such as dopaminergic degeneration and α-synuclein inclusions), Tao et al^102^ showed reduced *Sirt1* levels in the substantia nigra, a region particularly affected in the PD brain due to the loss of dopaminergic neurons.

A few studies have also explored specific groups of the KMT subclass of HMTs in AD mouse models. Cao et al^86^ reported elevated levels of *Kmt2a* and *SET-domain containing 1B histone-lysine methyltransferase* (*Setd1b)* in the prefrontal cortex of P301S Tau mice, with no significant change in *Kmt2b*, *Kmt2c*, *Kmt2d*, and *Setd1a*. However, in post-mortem prefrontal cortex from individuals with AD, the authors reported a different profile: increased levels of *KMT2C*, *KMT2D*, *SETD1A*, and *SETD1B*, but unaltered levels of *KMT2A* and *KMT2B* compared to control individuals.^86^ In another study, Zheng et al^85^ reported increased *euchromatic histone-lysine methyltransferase 1* (*Ehmt1)* and *2* (*Ehmt2)* levels in the prefrontal cortex of 5xFAD mice. Notably, the upregulation of *EHMT1* levels was also observed in human AD prefrontal cortical tissues in this study, although no significant change in *EHMT2* levels was found.^85^ These studies need to be reproduced in other AD mouse models and in humans in order to define the holistic changes in these enzymes and the implications of their dysregulation in AD and other neurodegenerative diseases.

## MicroRNAs

Regulatory non-coding RNAs (ncRNAs) comprise transcripts that are not translated into proteins and are key players in gene regulation instead.^103^ Their main role is the post-transcriptional regulation of gene expression, by affecting the stability and degradation of messenger RNA (mRNA), preventing its translation into proteins. ncRNAs can be categorized into short (<200 nucleotides) and long ncRNAs (>200 nucleotides). This review focuses on one specific subset of small ncRNAs—microRNAs (miRNAs)—which are approximately 21 to 22 nucleotides in length. Changes in brain-enriched miRNAs have been identified in many neurodegenerative diseases, including in studies using animal models, that will be discussed in the next sections.^103^

### Genome-wide microRNA profiling

Microarrays and sequencing techniques have been invaluable for the detection of dysregulated miRNAs in animal models of AD and PD.

Various studies have performed microarray analyses in the brain of a range of mouse models of AD (Table 6), including Tg2576,^104,105^ APP23,^106^ APP/PS1,^107-110^ 5xFAD,^111,112^ 3xTg-AD,^113^ SAMP8,^45,114-116^ senescence-accelerated mouse prone 10 (SAMP10),^116^ and PSEN dKO^117^ mice. Some of these studies focused on specific miRNAs, whilst others performed detailed investigations of the cellular and molecular pathways affected and targeted by these miRNAs. Higaki et al^105^ demonstrated upregulation of members of the miR-200 family (miR-141, -200a, -200b, -200c, -429) and miR-183 family (miR-96, -182 and -183) in the cortex of 10-month-old Tg2576 mice. Intriguingly, the upregulation of members of the miR-200 family in Tg2576 mice appeared to be limited to the phase of increasing Aβ plaque deposition. miR-200 family members have been shown to regulate neuronal proliferation, homeostasis and apoptosis, and hence their dysregulation may interfere with these vital regulatory processes.^105^ Liu et al^107^ reported downregulated miR-200a, -200b, -182, and -183, in contrast to some of the results reported by Higaki et al^105^ It is worth noting that the tissue samples were much different between the 2 studies; Liu et al^107^ investigated the hippocampus, whereas Higaki et al^105^ looked at cerebral cortex. Another study profiled miRNAs in APP/PS1 mice (whole brain) at 1-, 3-, 6-, and 9-months of age, to determine miRNA expression patterns over development and age.^108^ The changes in expression of some miRNAs overlapped between age groups, suggesting that these miRNAs may act across different stages of the disease. Some additional studies investigating Aβ-induced rat models of AD^118,119^ and an Aβ-induced *Drosophila* model,^120^ shared similar findings, including changes to neuronal health and vital signaling and regulatory mechanisms such as PI3K/Akt and Jak-STAT cellular pathways.

**Table 6.** MicroRNA microarray studies in animal models of Alzheimer’s disease and Parkinson’s disease.

Study	Model system	Age (months)	Brain tissue	Findings	Focus/Implications of study
Alzheimer’s disease					
Wang et al^104^	Mouse, Tg2576	12	HP	Several dysregulated miRNAs (eg, miR-124, -125a, -125b)	Upregulated miR-124 associated with synaptic dysfunction and memory loss
Higaki et al^105^	Mouse, Tg2576	10	CTX	↑ 7 miRNAs > 2-fold ↓ 1 miRNA > 2-fold	Upregulated miR-200 and miR-183 family members
Schonrock et al^106^	Mouse, APP23	2, 7, 13	HP	Several dysregulated miRNAs (eg, miR-409-3p, -148b, -30c, -9, -21)	miRNA deregulation in hippocampal cultures paralleled results from APP23 mouse
Liu et al^107^	Mouse, APP/PS1	9	HP	↑ 15 miRNAs > 2-fold ↓ 13 miRNAs > 2-fold	Downregulated miR-135a and −200b involved in disease pathogenesis and as potential biomarkers of AD
Wang et al^108^	Mouse, APP/PS1	1, 3, 6, 9	Whole brain	Several dysregulated miRNAs at different age points (eg, miR-342-3p elevated at 1-, 6-, and 9-mo of age)	Eleven aberrantly regulated miRNAs that are conserved in humans and are predicted to be associated with disease pathology, MAPK and TGF-β signaling pathways
Li et al^109^	Mouse, APP/PS1	5	HP	↑ 5 miRNAs ↓ 15 miRNAs	Upregulated miR-574 associated with synaptic plasticity
Wang et al^110^	Mouse, APP/PS1	3, 6	CTX	↑ 20 miRNAs ↓ 17 miRNAs	Upregulated miR-34a associated with apoptosis that may contribute to AD pathogenesis
Noh et al^111^	Mouse, 5xFAD	4, 8	HP	Several dysregulated miRNAs (eg, miR-139, -340, -3470a)	miR-139, -340 and −3470a associated with bio-energy metabolism related genes and metabolic dysfunction
Zhang et al^112^	Mouse, 5xFAD	4	HP	↑ 21 miRNAs ↓ 6 miRNAs	Downregulated miR-188 associated with disease neuropathology, neuroinflammation, and synaptic and cognitive impairments
Barak et al^113^	Mouse, 3xTg-AD	4, 16	HP	Several dysregulated miRNAs in both age groups (eg, miR-15a, -34a, -298, -101a, -294)	Predicted pathways upregulated include renal cell carcinoma, colorectal cancer, chronic myeloid leukemia, and glioma Predicted pathways downregulated include MAPK signaling, axon guidance and regulation of actin cytoskeleton
Cosín-Tomás et al^45^	Mouse, SAMP8	2, 9	HP	Several dysregulated miRNAs of which 6 miRNAs overlapped at both ages	Predicted pathways affected include oxidative stress, inflammation, pathological development, cell cycle dysregulation, neurogenesis
Zhou et al^114^	Mouse, SAMP8	3	HP	↑ 7 miRNAs > 1.5-fol*d* ↓ 8 miRNAs > 1.5-fol*d*	Downregulated miR-181c associated with regulation of axon guidance and MAPK signaling among other functional processes
Zhang et al^115^	Mouse, SAMP8	8	HP	Several dysregulated miRNAs (eg, miR-214-3p, 464-5p, -194-5p, −129a)	Downregulated miR-214-3p associated with autophagy and apoptosis
Zhang et al^116^	Mouse, SAMP8	3	HP	↑ 148 miRNAs ↓ 171 miRNAs	Co-upregulated miRNAs associated with MAPK, insulin and neurotrophin signaling pathways, and regulation of actin cytoskeleton
	Mouse, SAMP10	3	HP	↑ 139 miRNAs ↓ 163 miRNAs	
Ham et al^117^	Mouse, PSEN dKO	7, 12, 18	CTX, HP	Several dysregulated miRNAs in both brain regions	Age-dependent miRNAs in PSEN dKO mice and associated pathways indicate pathological aging
Wang et al^118^	Rat, Aβ_1-42_-induced	Age not stated	HP	↑ 93 miRNAs > 2-fold ↓ 90 miRNAs > 2-fold	Regulatory interactions between miRNAs and circular RNAs may play important roles in AD pathogenesis
Li et al^119^	Rat, Aβ_25-35_-induced	3, 6	HP	Several dysregulated miRNAs, for example, miR-30b, -129-5p	Upregulated miR-30b associated with neuronal injury, neuronal loss and neuroinflammation
Kong et al^120^	Drosophila, Aβ_42_-induced	Adult flies, 16 days post-treatment	Brain	↑ 8 miRNAs ↓ 9 miRNAs	Predicted pathways affected include MAPK signaling, dorso-ventral axis formation, Jak-STAT signaling pathway
Parkinson’s disease					
Asikainen et al^121^	*C. elegans*, α-synuclein	Fourth larval stage		↑ 5 miRNAs ↓ 7 miRNAs	Aberrantly regulated miRNAs in all three *C. elegans* models suggest they may be involved in neuropathophysiological mechanisms in disease
	*C. elegans*, *cat-1* mutation	Fourth larval stage		↑ 2 miRNAs ↓ 3 miRNAs	
	*C. elegans*, *pdr-1* mutation	Fourth larval stage		↑ 2 miRNAs ↓ 1 miRNA	
Shen et al^122^	*C. elegans*, HASN^A53T^ OX	Fourth larval stage		↑ 18 miRNAs ↓ 13 miRNAs	3 miRNAs (cel-miR-1018, cel-miR-230-3p, and cel-miR-797-5p) were predicted to target orthologs of human *K07H8.2* and *SLC41A1*

Abbreviations: AD, Alzheimer’s disease; CTX, cerebral cortex; HP, hippocampus; Jak-STAT, Janus kinase-signal transducer and activator of transcription; MAPK, mitogen-activated protein kinase; miR or miRNA, microRNA; PD, Parkinson’s disease; TGF- β, transforming group factor β.

The up arrow (↑) represents upregulation or an increase in level. The down arrow (↓) represents downregulation or a decrease in level.

In PD, a study by Asikainen et al and colleagues investigated dysregulation of miRNAs in three *C. elegans* models bearing the human α-synuclein A53T mutation, or mutations within the vesicular catecholamine transporter (*cat-1*) or parkin (*pdr-1*) ortholog, reporting differential expression of several miRNAs in these models, including miR-64 and miR-65 families (Table 6).^121^ Another study investigated miRNAs in *C. elegans* overexpressing human mutant α-synuclein and reported the dysregulation of 3 miRNAs when comparing mutants (HASN^A53T^ OX) to controls expressing wildtype human α-synuclein (HASN^WT^ OX).^122^

miRNA-sequencing (miRNA-seq) is a powerful method for miRNA profiling, which is replacing the use of microarrays, particularly since it provides an unbiased investigation of all miRNAs.^123^ Three studies to date have conducted miRNA-seq to interrogate miRNAs in the APP/PS1 mouse model of AD (Table 7).^124-126^ Specifically, Luo et al^126^ used 2 sibling pairs (transgenic mice and wildtype littermate controls) to identify AD-associated miRNA dysregulation. High-throughput deep miRNA-seq has also been employed on a mouse model of PD bearing the human α-synuclein A53T mutation; the team reported dysregulation of specific miRNAs in the substantia nigra, including miR-144-5p, miR-200a-3p and miR-542-3p.^127^

**Table 7.** MicroRNA sequencing studies in animal models of Alzheimer’s disease and Parkinson’s disease.

Study	Model system	Age (months)	Brain tissue	Findings	Focus/Implications of study
Alzheimer’s disease					
Ma et al^124^	Mouse, APP/PS1	6, 9	CTX	↑ 12 miRNAs at 6 mo, 27 miRNAs at 9 mo ↓ 24 miRNAs at 6 mo, 29 miRNAs at 9 mo	Interactions between dysregulated miRNAs, circular RNAs and messenger RNAs may play important roles in AD pathogenesis
Li et al^125^	Mouse, APP/PS1	1, 3, 6, 9	CTX	Several dysregulated miRNAs across all age groups, for example, miR-80 reduced at 6- and 9-mo of age	Interactions between dysregulated miRNAs and messenger RNAs may play important roles in the progression of AD
Luo et al^126^	Mouse, APP/PS1	9	CTX	Several dysregulated miRNAs, for example, miR-99b-5p, 138-5p, 100-5p	Predicted pathways affected include axon guidance, PI3K/Akt signaling pathway and MAPK signaling pathway
Parkinson’s disease					
Mo et al^127^	Mouse, α-synuclein	12	Midbrain	↑ 32 miRNAs > 2-fold ↓ 25 miRNAs > 2-fold	Candidate miRNAs from miRNA sequencing results were screened in cerebrospinal fluid from PD patients to identify potential biomarkers for PD diagnosis

Abbreviations: AD, Alzheimer’s disease; CTX, cerebral cortex; MAPK, mitogen-activated protein kinase; miR or miRNA, microRNA; PD, Parkinson’s disease; PI3K/Akt, phosphatidylinositol 3-kinase/protein kinase B; SN, substantia nigra.

The up arrow (↑) represents upregulation or an increase in level. The down arrow (↓) represents downregulation or a decrease in level.

It is worth considering that miRNAs are specific to particular mechanisms within species, therefore it will be crucial to identify and confirm the human counterparts of miRNAs investigated in mice and other organisms. Furthermore, recognizing their associated pathways and targets can make the effective use of these findings in translational research achievable.

### Candidate microRNA studies

The vast number of studies found during the literature search for this review consisted of candidate miRNA studies. These studies explored specific miRNAs to identify and confirm their dysregulation in the brain in the context of disease and determine their role in the disease process, using primarily RT-PCR. RT-PCR-based approaches detect miRNAs with high sensitivity and specificity, and are often used to validate microarray expression data or to identify changes identified in post-mortem brain tissues.^123^ Supplemental Tables S3–S5 summarize studies published to date investigating the dysregulation of miRNAs in AD, PD and ALS, respectively, with the reported miRNAs categorized according to their associated biological processes and pathways. Of note, the pathways and mechanisms described to be affected by these miRNAs are well-known features and drivers of neurodegeneration. When interpreting these findings, it is also important to acknowledge that miRNAs have diverse roles and that their effects on downstream pathways are frequently interconnected.

Many miRNAs have been investigated across several studies with concordant and discordant findings between different diseases. For example, miR-34a,^128-131^ miR-146a,^132-136^ and miR-155^135-137^ have been reported to be upregulated in AD rodent models. MiR-146a has also been shown to be disrupted in the cortex of ALS mice, but downregulated instead.^138^ In the study, Gomes et al and colleagues suggest that this downregulation of miR-146a in ALS mice may be an early event preceding the upregulation of other inflammatory molecules and pathways at the symptomatic stage of disease.^138^ The upregulation of miR-146a in AD models, on the other hand, may be involved in neuroprotective mechanisms possibly trying to prevent detrimental neuroinflammation in latter stages of disease. Other mutual miRNAs across neurodegenerative diseases include miR-124 (downregulated in AD^139^ and PD^140^ models), miR-34a (upregulated in AD^128-131^ and PD^141^ models) and miR-19a (upregulated in AD^142^ and ALS^143^ models), suggesting their involvement in common neurodegenerative processes. Interestingly, research on the dysregulation of some of these miRNAs in neuroglial cells has also emerged, including one study which showed miR-146a overexpression switched microglia to its neuroprotective phenotype in vitro and in APP/PS1 mice,^144^ adding to the body of research confirming the substantial role of glial cells in neurodegenerative diseases.

## Caveats and future perspectives

Epigenetics in neurodegenerative diseases, particularly in vertebrate and invertebrate models, is a growing research field, with only limited work having been done to date, mostly focused in mouse models, which we described throughout this review. Additional model organisms, such as *Drosophila* and *C. elegans*, and their research potential, have also been discussed. Taken together, the research summarized in this review corroborates the utility of model organisms to study epigenetic regulation in the context of brain disease. There are, however, caveats associated with using these models to study the aforementioned complex diseases, and limitations of using model organisms should be addressed in studies utilizing them. Inconclusive reports to date using these models pose a major challenge in understanding the real importance of epigenetic mechanisms for neurodegenerative processes. The reasons why inconclusive findings have been reported—between different models and when comparing models to human post-mortem brains—are, at least in part, possibly related to whether a given model organism is the most suitable for the biological problem in question. Additional models that better recreate human disease are needed, which may offer the opportunity to overcome these challenges. Recent efforts have started to be put in place to improve existing mouse models in order to, for example, improve the modeling of as many aspects of AD as possible. For instance, Neuner et al and colleagues developed an AD-BXD mouse model by crossing 5xFAD mice with mice from the BXD genetic reference panel.^145^ Likewise, Yang et al^146^ crossed the APP/PS1 mouse model of AD with wild-derived strains of mice. The resultant strains improve genetic heterogeneity in the mouse models, consequently better illustrating the extensive genetic and epigenetic variation seen in humans. Furthermore, the MODEL-AD consortium is developing and rigorously characterizing the next generation of AD mouse models, aiming to develop models that closely reflect the sporadic late-onset human form of AD.^147^ These newer models offer a valuable opportunity to further investigate changes in genomic regulation in AD, and similar efforts for other neurodegenerative disorders should be pursuit.

Importantly, model organisms should be perceived as models, and the fact that they cannot recapitulate all aspects of brain disease should be acknowledged, considered, and embraced in studies making use of them. Model organisms do present many advantages (eg, short life span, tissue accessibility, testing for drug and molecular targets, powerful for functional validations and for understanding biological mechanisms and pathways), and their use should be focused on these strengths. Functional experiments in particular constitute some of the most powerful uses for these models. Indeed, functional studies successfully characterizing epigenetic changes identified in humans have started to emerge. As a follow up of DNA methylation and H3K4me3 changes identified in the gene *ANK1* in human AD brains,^50,148^ its *Drosophila* ortholog has been functionally characterized, including to determine interactions with tau and Aβ, as well as how it is involved in neurodegeneration and memory processes.^149^

One limitation specific to epigenetic research using model organisms is that DNA methylation exhibits organism-specificity for certain species, with non-CpG DNA methylation being restricted to vertebrates, which is something important to consider when using invertebrate models.^37^ Overall, low levels of overall DNA methylation are found in organism such as *C. elegans*, and *D. melanogaster*. Despite *Danio rerio* (also known as zebrafish) being a well-established vertebrate model organism used in neuroscience, which exhibits DNA methylation,^150^ we did not come across any work to date using this powerful model to study DNA methylation in the context of neurodegenerative processes; future studies should explore genomic regulation processes in zebrafish models of neurodegenerative diseases.^18,37^ Similarly, non-human primates have many similarities to humans, including the presence of DNA methylation and the development of natural neurodegenerative pathologies^17^; very few studies have investigated genomic regulation in non-human primates, however, possibly as a result of the challenges associated with investigating non-human primates, such as their long lifespan, as well as ethical constrains in many countries. In fact, only one study to date investigated epigenetic changes in these models.^151^ Recently, Sato et al^152^ explored the generation of non-human primate models of AD to overcome some of their lifespan limitations, by adding mutations identified in familial cases of AD. The team focused primarily on the common marmosets (*Callithrix jacchus*), which exhibit much genetic, physiological, and anatomical proximity to humans. Most importantly, marmosets develop natural senile Aβ plaques and phosphorylated tau pathology in the brain. By inserting genetic mutations, as in other animal models discussed in this review, Sato et al^152^ reported that this fastens disease onset, facilitating the use of marmosets to study AD. These and additional, newly developed, models of AD, PD, and ALS are described in Table 8. Future studies should explore neuroepigenetic mechanisms in these models.

**Table 8.** More recent animal models of Alzheimer’s disease and Parkinson’s disease.

Study	Animal	Model	Description
Alzheimer’s disease			
Neuner et al^145^	Mouse	AD-BXD	5xFAD mouse model of AD crossed with mice from the BXD genetic reference panel
Yang et al^146^	Mouse	CAST.APP/PS1, WSB.APP/PS1, PWK.APP/PS1	APP/PS1 mouse model of AD crossed with multiple wild-derived strains of mice (CAST, WSB, or PWK)
Baglietto-Vargas et al^153^	Mouse	hAβ-KI	Knock-in of wildtype human Aβ under control of mouse App locus
Sato et al^152^	Common marmosets	PSEN1-ΔE9	Deletion of exon 9 of *PSEN1* gene
Dong et al^154^	Zebrafish	7 dpf psen1^Q96_K97del^/ + larvae	Deletion of 6 nucleotides in the zebrafish *psen1* gene
Barthelson et al^155^	Zebrafish	*psen2^T141 _ L142delinsMISLISV^* *psen2^N140fs^*	In-frame mutation or frameshift mutation, respectively, introduced at zebrafish *psen2* gene
Benbow et al^156^	*C. elegans*	Aβ_1-42_; tau-Tg	Pan-neuronally expresses both the toxic Aβ_1-42_ peptide and the wildtype 4R1N isoform of human tau
Parkinson’s disease			
Dave et al^157^	Rat	Pink1 KO, DJ-1 KO, Parkin KO	Knockout of Pink1, DJ-1, or Parkin genes
Pütz et al^158^	Drosophila	*Mbt*-null	Knockout of PAK4 homolog Mushroom bodies tiny

Abbreviations: AD, Alzheimer’s disease; dpf, days post-fertilization; KI, knock-in; KO, knock-out; Tg, transgenic.

Additional strategies that can mitigate some of the limitations associated to the use of animal models include the use of human induced pluripotent stem cells (iPSC)-derived brain cells (eg, iPSC-derived neurons), including in co-culture systems containing neurons and other major brain cell types (eg, astrocytes and microglia).^159^ A major advantage of using iPSCs is the fact that it offers the possibility of generating brain cells from any donor, including from sporadic cases of neurodegenerative diseases and even prodromal or asymptomatic individuals. These attractive technologies come with their own limitations, however; for example, as a consequence of their cellular reprograming, involving epigenetic remodeling, iPSC-derived brain cells are transcriptionally and epigenetically similar to immature brain cells.^160^ This makes them great for studying neuronal development but poses challenges when studying aging-associated diseases. Studies using iPSCs have already contributed greatly to our understanding of certain aspects of neurodegenerative diseases, and, in parallel to studies using animal models, can be very powerful in understanding and validating findings in mice. Indeed, 2 studies from the same laboratory explored DNA methylation changes in the hexanucleotide repeat expansion in the C9orf72 gene known to cause ALS, in human iPSCs and mice. Specifically, Esanov et al and colleagues reported that hypermethylation in the C9orf72 promoter, seen in some ALS patients, was recapitulated in motor neuronal differentiation in 1 iPSC line and in the cortex of only a subset of C9BAC mice similarly to what is observed in the human C9-ALS population.^161,162^ According to the study using iPSCs, 5mC levels are reduced with reprograming but re-acquired with differentiation into motor neurons.^161^ In addition to methylation changes in the C9orf72 promoter, the second study provides evidence that hypermethylation of the hexanucleotide repeat itself in the cortex of C9BAC mice increases with age, which aligns with the developmental progression seen in patients.^162^ Important limitations of these 2 publications by Esanov et al should be considered when interpreting their findings, however; examples include the investigation of a very low number of subjects (human and mouse), and, importantly, the observation of C9orf72 promoter hypermethylation with differentiation in motor neurons from a single iPSC line, as well as the use of a single mouse model. Further studies are needed to validate these findings, particularly to disentangle the complex relationship between 5mC and 5hmC in the context of C9orf72 repeat expansion.

Considering the research covered in this review, a limited number of studies explored enrichment of epigenetic marks to specific functional regions systematically. The study by Gjoneska et al and colleagues, profiling histone modifications in p25-inducible transgenic mice, explored functional enrichment of regulatory elements for changes in promoter and enhancer regions in detail.^83^ By performing enrichment analysis of gene ontology (GO) categories from differential gene expression analysis in the same mice, Gjoneska et al^83^ reported that increased-level enhancers and promoters were enriched for immune and stimulus-response functions, and decreased-level enhancers and promoters were enriched for synapse and learning-associated functions in CK-p25 mice. Enrichment of regulatory motifs uncovered distinct regulatory motifs for promoters and enhancers: increased-level peaks exhibited enrichment for NFκB, E2F, PPARG, IRF and PU.1 for both (suggesting targeting of immune regulation), decreased-levels in enhancers enriched for DNA-binding RFX motifs, and decreased-levels in promoters enriched for zinc-finger ZIC motifs. In addition to correlating histone marks with gene expression, the authors went even further by evaluating the effect of increased-levels for enhancer regions in gene expression in cell models and found that 8 out of the 9 increased-level human orthologs tested were indeed able to drive *in vitro* expression. Also at a functional validation level, Sanchez-Mut et al^66^ used a custom DNA methylation array specifically containing promoters of genes related to sensory perception, cognition, neuroplasticity, brain physiology and mental disorders, to interrogate relevant regulatory elements in 12 brain regions of APP/PS1 and 3xTg-AD mice. Importantly, hypermethylation of promoter regions for *Sorbs3*, *Spnb4* and *Tbxa2r* in the prefrontal cortex of APP/PS1 and 3xTg-AD mice was also observed in human frontal cortex (corresponding human orthologs) from individuals at late stages of AD.

One major methodological caveat of DNA methylation studies to date is the use of sodium bisulfite treatment as part of the laboratory techniques to profile DNA methylation, which hinders the ability to differentiate between 5mC and 5hmC.^163^ This is important because 5hmC is an individual methylation mark alongside 5mC,^164^ which regulates many relevant brain processes, as mentioned. Oxidative bisulfite conversion can be employed to overcome this issue, where 5hmC is converted to 5fC, and then uracil, thus allowing accurate detection of 5mC alone; 5hmC can be estimated from the quantification of the difference between bisulfite and oxidative bisulfite conversions.^165^ It should be noted that any approach involving bisulfite conversion damages nucleic acids, resulting in short DNA fragments, however. The uprising of third-generation sequencing (also known as long-read sequencing) technologies, such as nanopore sequencing, provide encouraging alternatives that can overcome limitations of conventional bisulfite sequencing. Despite the excitement associated with the opportunities that forefront sequencing technologies can offer, including next-generation sequencing in addition to long-read sequencing, these methods are realistically not accessible to all research laboratories, mostly because of their high costs, the need for specialized equipment and staff, and the scarcity of standardized bioinformatic pipelines for epigenomic analyses. Methylation arrays are reliable and still widely used alternatives for the interrogation of selected methylation sites across the genome, offering reduced costs, i.e., proving more accessible and feasible for many research groups. Vertebrate arrays, and in particular standardized mouse methylation arrays, also offer insightful venues for studying DNA methylation, and should also be considered for near future studies.

Of great importance, studies in model organisms of neurodegenerative diseases performed so far have not explored epigenetic changes in specific brain subregions and their layers, and in different cell populations. Firstly, different brain regions and their subregions are involved in controlling a diverse range of activities and behaviors and are affected differently in disease. Most importantly, different tissues and cell types have distinct epigenetic profiles, which regulate their unique functions. A range of cells, from neurons to glial cells (such as microglia, astrocytes, and oligodendrocytes), and their respective subtypes, make up the cells in the brain, each with important individual roles. The use of cell deconvolution computational algorithms enables the opportunity to disentangle, at least in part, cell-specific patterns and proportions in studies using bulk samples, improving interpretability and reducing confounding effects of cellular heterogeneity. These computational techniques rely on reference panels and their quality, accuracy, and similarity to the testing samples, however, often revealing not to be the most appropriate approach to employ. The purification of different cell populations using cell sorting techniques, such as fluorescence-activated cell sorting,^166^ magnetic affinity cell sorting^167^ and laser capture microdissection, can overcome these limitations.^168^ Additionally, emerging single-cell technologies offer a powerful and reliable space for refining epigenomic regulation at the level of individual cells, allowing the identification of key individual cell changes and how different cell and their associated changes relate to each other, as well as the identification of relevant cell populations. Combined with the ongoing rise of spatial epigenomics, rendering the possibility to survey distributions in different regions, layers, and cells of the brain, the fast-growing field of epigenomics (and multiomics) technologies promises many breakthroughs and much knowledge expansion in the next few years. Moving forward, it is important that future epigenetic studies discriminate cell-specific profiles, and thus take advantage of state-of-the art methodologies, such as the above-mentioned technologies, to collect more refined and insightful data for the better understanding of neurogenerative diseases and their complexity.

Epigenetic research has gained a lot of attention in recent years, with the implication of epigenetic processes in the brain, and particularly in neurodegenerative diseases, having been an important contributor in better understanding these conditions. Of note, epigenetic mechanisms are dynamic and reversible, changing throughout life, development, aging and disease, and being influenced by the environment. Importantly, this also implicates that they possess high potential as disease biomarkers and drug targets.^16^

As technologies continue to advance and models continue to improve, more studies taking advantage of cutting-edge laboratory and analytical approaches to survey model organisms and their overlaps with humans are necessary, to grasp a better understanding of how and when we should modify and manipulate aspects of human neurodegenerative diseases that are recapitulated in these models.

## Conclusions

Research investigating neurodegenerative diseases, including AD, PD, and ALS, has shown that genetics alone cannot explain disease etiology, and that additional genomic processes, such as epigenetic mechanisms, may also have an important contribution. Understanding the role of these mechanisms in said diseases is still a nascent research field, and more is still to be understood about the consequences of any changes in the expression and activity of epigenetic regulation.

Human studies exploring the epigenetic landscape of brain diseases rely on post-mortem tissue, which usually corresponds to end stages of the disease and often displays significant degeneration, hence the use of model organisms offers the advantage to in understand epigenetic changes at different stages of the neurodegenerative process, including at earlier stages, as well as changes that mirror disease progression.

Throughout this review we discussed a range of vertebrate and invertebrate models that have been used to date to model neurodegenerative diseases to investigate epigenetic changes, which has mostly focused on studying AD. In parallel with advancements of technologies and laboratory methods for epigenomic assessments, increasing efforts in developing innovative and viable disease models are currently underway; it will be paramount to maximize the use of these models to ascertain the genomic dysregulation in neurodegenerative diseases, in order to identify and test effective drug targets and molecules for treating them (Figure 1). Future research expanding on the studies described here, especially studies taking advantage of emerging single-cell and spatial epigenomic technologies, will certainly transform our understanding of neurodegenerative diseases and deliver important insights that can be applied for their alleviation.

## Supplemental Material

sj-docx-1-gae-10.1177_25168657221135848 – Supplemental material for The Neuroepigenetic Landscape of Vertebrate and Invertebrate Models of Neurodegenerative Diseases Click here for additional data file. Supplemental material, sj-docx-1-gae-10.1177_25168657221135848 for The Neuroepigenetic Landscape of Vertebrate and Invertebrate Models of Neurodegenerative Diseases by Thanga Harini Sundaramoorthy and Isabel Castanho in Epigenetics Insights
