# Supplementary material for: The Neuroepigenetic Landscape of Vertebrate and Invertebrate Models of Neurodegenerative Diseases
Source: Epigenet Insights. 2022 Nov 4;15:25168657221135848. doi: 10.1177/25168657221135848 (PMC9638687; doi:10.1177/25168657221135848)
Supplement: sj-docx-1-gae-10.1177_25168657221135848 – Supplemental material for The Neuroepigenetic Landscape of Vertebrate and Invertebrate Models of Neurodegenerative Diseases [file sj-docx-1-gae-10.1177_25168657221135848.docx]

**Supplementary Tables**

**Table S1:** Literature search terms and strategy

|  | Search Terms |
| --- | --- |
| Population | “mouse” OR “mice” OR “rats” OR “rodent” OR “Drosophila” OR “fly” OR “zebrafish” OR “Danio rerio” OR “C. elegans” OR “monkey” OR “primate” OR “animal model” OR “in vivo” OR “vertebrate” OR “invertebrate” |
|  | AND |
| Disease | “Alzheimer’s” OR “Parkinson’s” OR “amyotrophic lateral sclerosis” OR “ALS” |
|  | AND |
| Epigenetic marks and enzymes | “DNA methylation” OR “5mc” OR “5hmc” OR “DNMT” OR “TET” OR “histone modification” OR “histone methylation” OR “histone acetylation” OR “histone phosphorylation” OR “H3K*” OR “H4K*” OR “HMT” OR “HAT” OR “HDAC” OR “SIRT” OR “microRNA” OR “miRNA” OR “epigenetics” |

* denotes variable suffixes that are screened by the database and included in the search.

**Table S2:** Inclusion and exclusion criteria for primary literature

| Inclusion criteria | Exclusion criteria |
| --- | --- |
| - Studies investigating animal models of AD, PD and ALS - Studies investigating any of the following epigenetic mechanisms: DNA methylation, histone modifications, microRNAs - Studies investigating brain regions - Studies that include a control group - All publication dates - All geographical locations - Available in English language | - Studies that do not fulfil the inclusion criteria - Review articles - Dissertations and theses - Retracted articles |

Abbreviations**:** AD, Alzheimer’s disease; ALS, amyotrophic lateral sclerosis; PD, Parkinson’s disease.

**Table S3:** Dysregulation of candidate miRNAs in the brain in rodent models of Alzheimer’s disease

| microRNA | Associated pathway(s) | Reference |
| --- | --- | --- |
| Upregulated / Increase in level | |  |
| miR-19a-3p | Synaptic function | ^142^ |
| miR-26a-5p | Synaptic function | ^142^ |
| miR-26b | Aβ deposition | ^169^ |
| miR-30b | Synaptic function | ^98^ |
| miR-34a | Synaptic function, Aβ deposition | ^128-131^ |
| miR-125b-5p | Synaptic function | ^142^ |
| miR-128 | Aβ deposition | ^170^ |
| miR-136-3p | Synaptic function | ^142^ |
| miR-139 | Neuroinflammatory processes | ^171^ |
| miR-140 | Autophagy, mitochondrial dysfunction | ^172^ |
| miR-145 | *Not certain from study* | ^136^ |
| miR-146a | Neuroinflammatory processes (increase in NF-κB, cytokine production), glial cell activation, tau phosphorylation, Aβ deposition | ^132-136^ |
| miR-155 | Neuroinflammatory processes (cytokine production) | ^135-137^ |
| miR-181 | Synaptic function | ^99^ |
| miR-200a-3p | Neuronal apoptosis | ^100^ |
| miR-204 | Autophagy, mitochondrial dysfunction | ^173^ |
| miR-206-3p | Brain-derived neurotrophic factor expression | ^174^ |
| miR-219 | *Not certain from study* | ^99^ |
| miR-455-5p | Synaptic function | ^175^ |
| miR-708 | *Not certain from study* | ^99^ |
| Downregulated / Decrease in level | |  |
| miR-9 | Aβ deposition, cognitive function | ^176^ |
| miR-15 | *Not certain from study* | ^177^ |
| miR-29c-3p | Wnt/β-catenin pathway | ^178^ |
| miR-124 | Aβ deposition, angiogenesis | ^139^ |
| miR-130a-3p | Cognitive function | ^179^ |
| miR-135a-5p | Synaptic function | ^142^ |
| miR-137 | Tau phosphorylation | ^180^ |
| miR-153 | Aβ deposition | ^181^ |
| miR-181a | Aβ deposition, cognitive function, pericyte apoptosis | ^182^ |
| miR-188-5p | Synaptic function, cognitive function | ^183^ |
| miR-195 | Aβ deposition | ^177^ |
| miR-196a | Apoptosis, oxidative stress injury, PI3K/Akt pathway | ^184^ |
| miR-218 | *Not certain from study* | ^99^ |
| miR-222 | Cell cycle regulation | ^185^ |
| miR-338-5p | Aβ deposition, glial cell activation, synaptic function, cognitive function | ^186^ |
| miR-384 | Expression of amyloid precursor protein (associated with Aβ deposition) | ^187^ |
| miR-485-5p | Pericyte viability and apoptosis | ^188^ |
| miR-873-5p | Neuronal apoptosis | ^189^ |
| Mixed findings | |  |
| miR-16 | *Uncertain from studies to date* | ^136,177^ |
| miR-29c | Expression of neurone navigator 3 (a regulator of axon guidance), Aβ deposition | ^190,191^ |
| miR-106b | TGF-β signalling pathway, neuronal apoptosis | ^192,193^ |
| miR-132 | Cognitive function, tau phosphorylation, oxidative stress | ^194-197^ |

Abbreviations: NF-κB, nuclear factor-κB; PI3K/Akt, phosphatidylinositol 3‑kinase/protein kinase B; TGF-β, transforming growth factor-β

**Table S4:** Dysregulation of candidate miRNAs in the brain in rodent models of Parkinson’s disease

| microRNA | Associated Pathways | Reference |
| --- | --- | --- |
| Upregulation / Increase in level | |  |
| miR-21 | Expression of α-synuclein | ^198^ |
| miR-34a | *Not certain from study* | ^141^ |
| miR-103a-3p | Mitophagy | ^199^ |
| miR-204-5p | Expression of α-synuclein, autophagy, apoptosis | ^200^ |
| miR-342-3p | *Not certain from study* | ^201^ |
| miR-384-5p | Apoptosis | ^102^ |
| Downregulation / Increase in level | |  |
| let-7e | *Not certain from study* | ^202^ |
| miR-7 | *Not certain from study* | ^141^ |
| miR-10a | Apoptosis | ^203^ |
| miR-23b-3p | Modulation of PD-related genes, such as α-synuclein gene | ^201^ |
| miR-26b | *Not certain from study* | ^202^ |
| miR-30b-5p | Modulation of PD-related genes | ^201^ |
| miR-30e | Expression of α-synuclein, neuroinflammatory processes (cytokine production, inflammasome activity) | ^204^ |
| miR-34b-5p | Oxidative stress | ^205^ |
| miR-124 | Cell cycle regulation | ^140^ |
| miR-125b-5p | Autophagy, apoptosis | ^206^ |
| miR-128-3p | Neuronal apoptosis | ^207^ |
| miR-132-3p | Neuroinflammatory processes (cytokine production), glial cell activation | ^208^ |
| miR-141 | *Not certain from study* | ^202^ |
| miR-195-3p | Modulation of PD-related genes | ^201^ |
| miR-214 | Expression of α-synuclein | ^209^ |
| miR-218-5p | Apoptosis, oxidative stress | ^210^ |
| miR-326 | Apoptosis, oxidative stress | ^211^ |
| miR-425 | Necroptosis | ^212^ |
| miR-599 | Modulation of *LRRK2* gene expression | ^213^ |
| Mixed findings |  |  |
| miR-26a | Expression of α-synuclein | ^141,202^ |

Abbreviations: PD, Parkinson’s disease.

**Table S5:** Dysregulation of candidate miRNAs in the brain in mouse models of amyotrophic lateral sclerosis

| microRNA | Associated Pathways | Reference |
| --- | --- | --- |
| Upregulation / increase in level | |  |
| miR-9 | Regulation of neural fate | ^143^ |
| miR-19a | Cell cycle regulation | ^143^ |
| miR-19b | Cell cycle regulation | ^143^ |
| miR-124a | Regulation of neural fate | ^143^ |
| Downregulation / decrease in level | |  |
| miR-21 | Neuroinflammatory processes | ^138^ |
| miR-125b | Neuroinflammatory processes | ^138^ |
| miR-146a | Neuroinflammatory processes | ^138^ |
